# Co-Stimulatory Blockade of the CD28/CD80-86/CTLA-4 Balance in Transplantation: Impact on Memory T Cells?

**DOI:** 10.3389/fimmu.2015.00411

**Published:** 2015-08-10

**Authors:** Simon Ville, Nicolas Poirier, Gilles Blancho, Bernard Vanhove

**Affiliations:** ^1^Unité Mixte de Recherche, UMR_S 1064, Institut National de la Santé et de la Recherche Médicale, Nantes, France; ^2^Institut de Transplantation Urologie Néphrologie (ITUN), Université de Nantes, Nantes, France; ^3^Effimune SAS, Nantes, France

**Keywords:** CD28, CTLA-4, costimulation blockade, memory T cell, effector T cell, transplantation immunology, heterologous immunity, CTLA4-Ig

## Abstract

CD28 and CTLA-4 are prototypal co-stimulatory and co-inhibitory cell surface signaling molecules interacting with CD80/86, known to be critical for immune response initiation and regulation, respectively. Initial “bench-to-beside” translation, two decades ago, resulted in the development of CTLA4-Ig, a biologic that targets CD80/86 and prevents T-cell costimulation. In spite of its proven effectiveness in inhibiting allo-immune responses, particularly in murine models, clinical experience in kidney transplantation with belatacept (high-affinity CTLA4-Ig molecule) reveals a high incidence of acute, cell-mediated rejection. Originally, the etiology of belatacept-resistant graft rejection was thought to be heterologous immunity, i.e., the cross-reactivity of the pool of memory T cells from pathogen-specific immune responses with alloantigens. Recently, the standard view that memory T cells arise from effector cells after clonal contraction has been challenged by a “developmental” model, in which less differentiated memory T cells generate effector cells. This review delineates how this shift in paradigm, given the differences in co-stimulatory and co-inhibitory signal depending on the maturation stage, could profoundly affect our understanding of the CD28/CD80-86/CTLA-4 blockade and highlights the potential advantages of selectively targeting CD28, instead of CD80/86, to control post-transplant immune responses.

## Introduction

The importance of costimulation to allo-immune response has been widely demonstrated. A comparative study of xeno- and allo-immune response by Lafferty et al. in the late 60s was at the origin of this concept (1, 2). To their surprise, they found that “as the genetic relationship between donor and recipient becomes more distinct, the degree of reactivity falls to an undetectable level.” They proposed that something more than antigens, with species compatibility, was required to stimulate an allograft response. They called this second signal the allograft stimulus. This became the costimulation signal, when extended to the entire T-cell response, within the second signal theory (3).

Later, CTLA-4, an inhibitory cell surface molecule with the same ligand on antigen-presenting cell (APC) as CD28, namely CD80/86, was discovered, defining the CD28/CD80/86/CTLA-4 balance. This pathway became an attractive focus in the transplantation field and has been the target of much research over the past few decades, leading to the development of CTLA4-Ig (4). This fusion protein binds CD80 and CD86, preventing ligation of CD28 and also of CTLA-4. In spite of its proven effectiveness in inhibiting allo-immune responses, particularly in murine models, clinical experiences in kidney transplantation with belatacept (a high-affinity CTLA4-Ig molecule) have exhibited a high incidence of acute, cell-mediated rejection (5). The etiology of this belatacept-resistant rejection has been ascribed to heterologous immunity, i.e., the cross-reactivity of the pool of memory T cells from pathogen-specific immune responses, with alloantigens (6).

From the beginning, Lafferty et al. found that, once generated, activated cytotoxic T lymphocytes are able to kill any cell that expresses foreign antigens, that is, once activated the requirement for allogenic stimulus is lost (2). Based on a small number of studies, the idea that CD28 costimulation is unnecessary for CD4 + and CD8 + T-cell memory responses has become a generally accepted paradigm in immunology (7). This is consistent with the classic view that most T cells die after reacting to pathogens, but some of them, cells that are capable of destroying the pathogen, give rise to memory cells. Thus, the loss of the costimulation requirement is considered as a selective advantage to the memory T cells, which increases the efficiency of recall responses.

However, lines are shifting: in the memory field a new model, known as “developmental,” where naïve cells directly develop into memory cells without transitioning through an effector stage, is emerging. At the same time, data from experimental models, which are increasingly relevant to anti-infectious immune response, challenge the current paradigm of dispensable CD28 costimulation by memory T cells. Furthermore, advances in the field of cancer immunotherapy provide indication on the impact of CTLA-4 blockade, including on a preexisting immune response. This review delineates how this shift in paradigm could profoundly affect our understanding of the CD28/CD80/86/CTLA-4 blockade and highlights the potential advantages of selectively targeting CD28, instead of CD80/86, to control post-transplant immune responses.

## What We Can Learn from CD28-Negative T Cells

A way to investigate the requirement of CD28 for antigen-experienced T cells is to focus on CD28-negative T lymphocytes, for which there is little doubt that activation is CD28 independent.

As noted above, usually the loss of CD28, and consequently of the costimulation requirement, is regarded as a special state, achieved following an immune response by the most efficient clones and generating the best protective anamnestic response due to memory cells (8).

CD28-negative T cells are absent from umbilical cord blood, then emerge over time and finally a majority of peripheral blood T cells become CD28 negative (9). The loss of CD28 is an immunological feature primarily observed in humans and primates. Substantial progress has been made in understanding the molecular, cellular, and functional features of CD28-negative T cells since their initial identification in the early 90s (9, 10). On the one hand, they gain cytolytic activities supported by elevated expression of key molecules including perforin and granzyme, they have a low activation threshold, and in selected cases, render cell activation independent of the recognition of the appropriate antigenic peptide. On the other hand, they have a reduced capacity to proliferate and survive after TCR activation, displaying signs of lymphocyte exhaustion with dominant inhibitory receptors (8–10). Thus, their overall impact is negative, since their accumulation comes at the expense of an appropriate immune response and gives rise to the risk of autoimmunity.

CD28-negative T cells do not appear to be memory cells, whose function would be to improve a recall response, but terminally differentiated cells arising as a consequence of immune-senescence. This contradicts mainstream thinking, where loss of the costimulation requirement is considered as an advantage for memory cells.

## The Developmental Model and Possible Prediction of Costimulation Requirement

A new model for the linage relationship of T-cell subsets suggests that less differentiated memory T cells give rise to effector cells, and not vice versa, so memory cells are derived directly from activated naïve cells that have never experienced an effector state (11–13).

**Figure 1:**
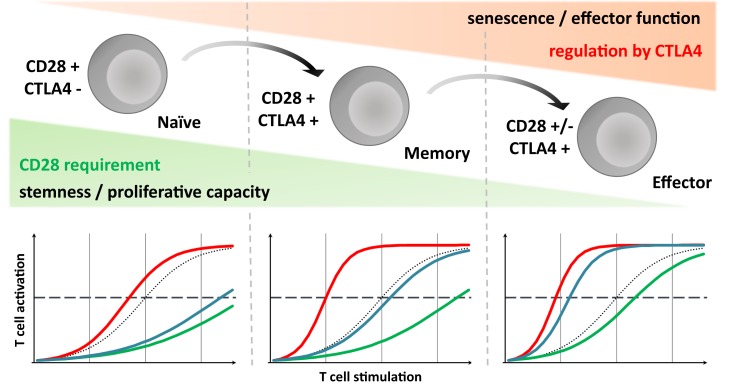
**CD28 requirement and CTLA-4 mediated inhibition evolve through T cell run, highlighting consequence of different strategies targeting the CD28/CD80/86/CTLA-4 axis**. Upper panel: according to the developmental model, during immune response, T cells differentiate progressively ranging from naive to effector via the memory stage. Throughout this process, like a ball rolling down a hill, they lose their proliferative potential but gain effector and homing competences. We assume that simultaneously their activation threshold, and so CD28 dependency, decreases but that conversely the importance of CTLA-4 intrinsic inhibitory signaling gradually increased. Lower panel: dotted line, control condition; red line, CTLA-4 blockade; green line, CD28 selective blockade; blue line, CD80/86 blockade; broken line represent sufficient level for T-cell activation and mounting an efficient response. For naive T cells, due to the lack of CTLA-4 signaling, selective and non-selective CD28 blockade would be equally efficient in controlling their activation. In the case of terminally differentiated T cells, preserving CTLA-4 mediated signals could be essential, especially in the absence of a CD28 requirement, suggesting a relevant advantage of the CD28 selective blockade compared to CD80/86 antagonist. Memory T cells might represent a middle path in which the intensity of the TCR stimulation, more important in allo-immune context especially with direct presentation, is probably critical.

This model is called developmental because it proposes that T-cell differentiation is largely a linear and unidirectional process, whose driving force is the cumulative history of antigenic stimulation, going from naïve cell to terminally differenced effector T cell via a memory stage, with progressive chromatin change. Cell maturation has been likened to a ball rolling down a hill, with cells progressively losing potential energy, i.e., “stemness” and proliferative capacities, but gaining effector and homing capacities (Figure 1). This process is associated with progressive characteristic changes in cell surface molecule expression that allow us to classify cells into various subsets. The loss of CD28 is one of the last events occurring during maturation (13). This fits with the features of CD28-negative T cells described above (9, 10).

CD28 loss, and from a broader perspective, loss of the costimulation requirement, would not then reflect an advantage inherent to memory acquired following an immune response, incidentally with a risk of immunopathology by inappropriate reactivation, but a feature of cells reaching the end of a progressive maturation process with limited potential but with effector capacities, and restricted to peripheral tissues.

## Origins of the Notion That Memory T Cells are Costimulation-Independent

The costimulation field has become much more complex since the publication of Lafferty’s allogenic stimulus hypothesis. Numerous activators and inhibitors of ligand/receptor interactions have been described on both sides of the immunological synapse and given the appellation cell surface signaling molecules. Their distribution is extremely variable according to their developmental stage, the localization of the different lymphocyte subsets and their propensity to impact on each other through feedback loops. The fate of each cell thus depends on the integration of signals derived from a large complex of stimulatory and inhibitory interactions (14). This framework, much more complex than the standard view of the second signal model, is required to interpret the results of the numerous studies that have been conducted over recent years on the CD28/CD80/86/CTLA-4 triad. Some conclusions, sometimes considered as “ground rules”, have to be tempered by the inevitable limitations of the particular experimental methodologies and model systems. One of these ground rules is the paradigm that memory T-cell activation is CD28 independent; an idea based on a small number of *in vitro* studies and ones on CD28-deficient mice.

CD28 signaling requirements in memory CD4 + and CD8 + T-cell responses have been much less well studied than those on primary response generation. A first experimental model used by Steinman 30 years ago was the mixed lymphocyte reaction (MLR) (15, 16). “Memory cells” resulting from primary MLRs were actually not true memory cells as defined today, but rather lymphoblasts. Unlike naïve T cells that proliferate only after stimulation with allogenic dendritic cells (DCs), these lymphoblasts proliferate regardless of the APC subset, including macrophage or B cell. The conclusion was that once activated, lymphocytes become independent of second signals.

These data were confirmed by Croft (17, 18). Adoptive transfer of TCR transgenic T cells, previously activated specifically *in vitro*, allowed exploration of antigen-specific memory responses. Indeed, after homeostatic proliferation in the host, they become memory-like, and, once harvested from spleen, they could be specifically re-activated *ex vivo* with specific peptides exogenously loaded onto various cultured APCs. Then using APC from CD80/86-deficient mice or CTLA4-Ig, the CD28-independence of these memory T cells was demonstrated (19, 20).

We should stress that all the previously discussed *in vitro* studies have examined CD28 costimulation requirements under conditions where the T-cell stimulus was not equivalent to the stimulus received in physiological conditions. Peptide was exogenously loaded onto cultured APCs, and thus the requirement for costimulation may have been overcome due to the strength of TCR signaling (21). Indeed, even for a primary response, the costimulation requirement can be overcome if sufficiently high levels of TCR stimulation are obtained. Viola et al., showed *in vitro* that, independent of the nature of the TCR stimuli, if TCR stimulation exceeds a minimum threshold, complete activation is achieved and in the presence of CD28 costimulation, that threshold is significantly lower (22), especially in memory T cells (23). Thus, the costimulation requirement is a quantitative phenomenon and has to be investigated in the light of the strength of TCR stimulation.

However, evidence *in vivo* was provided in a report by Suresh et al. showing that, in lymphocytic choriomeningitis virus (LCMV) infected CD28-deficient mice, memory LCMV-specific CD8 + T-cell response seems to be normally reactivated. Indeed when they were re-challenged with a lethal dose of LCMV, all the mice survived while all naive controls died (24).

At first sight, the use of CD28-deficient mice to investigate a memory response *in vivo* may seem questionable, since the primary response, and consequently the establishment of memory cells in these animals, is greatly reduced (25). But initial studies using LCMV-infected mice revealed that, unlike for principle viruses, an efficient primary CD8 + T-cell response can be generated in the absence of CD28 costimulation (25). The reason for this discrepancy was ascribed to higher levels of TCR stimulation, which could overcome the need for costimulation. Therefore, using this model to explore the recall responses actually makes little sense. In addition, more detailed studies suggest a number of deficiencies in terms of the primary LCMV-specific T-cell response in CD28-deficient mice. In particular, the expansion of virus-specific CD4 T cells was reduced by about a factor of 10 (26) and results with B7-deficient mice indicate that B7 costimulation is required for induction and maintenance of LCMV-specific CD8 + T-cell memory (27). Finally, although CD28-deficient mice have normal levels of B- and T-cell populations, given the importance of CD28 costimulation in thymic T-cell development (28, 29), lack of CD28 induces a defect in regulatory T cells and could lead to defective mature T cells. Taken together, this complicates using these mice to study memory responses.

In the early 2000s, based on *in vitro* studies and models of LCMV infection in CD28-deficient mice, despite their methodological and technical limitation, the prevailing belief was that CD28 costimulation was not required for memory T-cell activation.

## Revisiting the Concept

The accepted hypothesis began to be questioned with studies (30, 31) in which *in vivo* depletion systems were used to analyze the role of DCs in reactivating CD8 memory T cells during recall response to three different microbial infections. Without DCs during recall response, a profound decrease in the number of CD8 memory T cells was observed, suggesting that costimulation through DCs was required for optimal memory response.

More evidence against the proposition that costimulation is dispensable for memory T cells comes from successes in the treatment of autoimmune diseases, which by definition involve pre-existing auto-reactive T cells. CTLA4-Ig has proved effective both in experimental models (32, 33) and in the clinic with psoriasis (34) and rheumatoid arthritis (35).

Furthermore, except for the specific case of LCMV infection, a lack of costimulation by CD28 impaired secondary response in numerous other infectious models has been found (36–42). Whether these observations indicate requirements for CD28 costimulation during the initial priming or during the recall response is not clear and has not been investigated in detail.

In several more recent works (27, 43–50), reactivation of specific memory T-cell populations in immunized WT mice has been investigated using specific tetramers, or by adoptive transfer of labeled memory T cells. Assessment of a CD28 requirement was made through either adoptive transfer in CD80/86-deficient hosts or through costimulation blockade at the moment of recall, using CTLA4-Ig or anti-CD28 antagonist antibodies.

The essential function of CD28 for conferring host protection during secondary infection has been confirmed using the cre-lox system allowing a CD28-inducible KO in a model of infection by N. Brasiliensis (51). Mice were infected a first time, then a second one after treatment with tamoxifen allowing an efficient CD28 deletion. Compared with WT, these mice had a delayed expulsion of adult worms in the small intestine.

Finally, a more recent study highlighted the critical importance of the CD28 pathway to memory T-cells homeostasis (52). Again in a context of LCMV infection, Kalia et al. demonstrated that without Tregs, memory T cells in a quiescent state proliferated and engaged terminal differentiation. CTLA-Ig by blocking CD80/86 interaction with CD28 rescued memory defects (maintaining a quiescent state) by mimicking Treg known to modulate the level of ligand available for CD28 through CD80/86 trans-endocytosis on APC mediated by CTLA-4 (53).

Thus, currently, extensive research using more relevant experimental models has demonstrated that the optimal elaboration of secondary T-cell immunity, as well as memory T-cell homeostasis, is dependent on productive CD28/CD80/86 interactions, in the setting of anti-infectious immune response.

## Allo-Immune Response

As a starting point, we have to distinguish two dramatically different scenarios for the involvement of immune memory response in transplantation. First, recipients who are sensitized to HLA antigens, which occurs mainly through blood transfusions, pregnancy, or previous organ transplantation (54–56). To date, very little research has been done on use of T-cell-specific costimulation blockade strategies in HLA-sensitized recipients and as such it will not be addressed in this review. Second, there are recipients without HLA-specific immunization. In such case, memory T-cell involvement is not, at first sight, obvious.

In the early 90s, shortly after its discovery, the CD28/CD80/86 interaction blockade, later associated with CD40–CD40L blockade, raised great hopes in the transplantation field. In murine models, numerous studies demonstrated that blockade of these co-stimulatory pathways during transplantation was highly effective at tolerizing naive donor-reactive T cells and prolonging graft survival. This occurred irrespective of the blockade modality: CTLA4-Ig (57–59) or anti-CD80/86 antibodies (4). While treatment with CTLA4-Ig in rodents demonstrated high efficacy, experiments in non-human primates demonstrated much more modest prolongation of allograft survival (60–62).

Initially, a weak affinity of the first CTLA4-Ig for CD86, compared with CD80, was hypothesized as the source of this lack of effectiveness (4). Thus, a second generation of CTLA4-Ig, LEA29Y, with a better affinity for CD86, was developed. Translation of LEA29Y into non-human primate models of renal transplantation showed superior prolongation in graft survival compared to CTLA4-Ig as a monotherapy, and dramatically improved survival when used as part of a combined immunosuppressive regimen including either mycophenolate mofetil (MMF) and steroids or anti-IL-2R (basiliximab) (63). Based on these encouraging results, LEA29Y (belatacept) was moved into clinical trials as the principal component of an immunosuppressive regimen consisting of basiliximab, steroids, and MMF (5). As expected, this study showed improvement in renal function compared with cyclosporine-treated recipients owing to reduced CNI-related renal toxicities (5, 64). However, the incidence of biopsy-proven acute rejection was higher in belatacept-treated recipient, giving rise to a new concept: the “belatacept-resistant rejection,” its counterpart being resistance to tolerance induction in rodent experimental models.

As detailed above, based on studies *in vitro* and in CD28-deficient mice, the perception that memory cells did not require costimulation signaling by CD28 was deeply ingrained. Consequently, memory T cells were presumed to be the guilty party in belatacept-resistant rejection via heterologous immunity, the concept that without bystander activation, virus-specific memory T cells can become activated by unrelated viruses, through molecular mimicry (65). On the top of this, unexpected cross-reactivity between virus-specific CTL clones and uninfected allogenic targets has been demonstrated (66). This activity could be attributed to dual recognition of pathogen-peptide/self-CMH complexes as well as peptide/allo-CMH complexes (6, 67). The most famous example is in seminal studies by Burrows et al. demonstrating that CD8 + T cells specific to EBV-EBNA3A restricted by HLA-B8 were cross-reactive with HLA-B44 presenting a self-peptide. Recently, the molecular understanding of this phenomenon has improved (68, 69) and its magnitude in the transplantation context has been clarified (70).

Heterologous immunity was suspected of playing a major role in mediating costimulation blockade-resistant allograft rejection, observed in situations where transplant recipients have an immune history. Several studies argue for this hypothesis, showing that naive recipients that had previously been infected with different pathogens became refractory to the tolerance-inducing effects of costimulation blockade (71, 72). This resistance is transmitted by adoptive transfer of CD8 and/or CD4 from an immunized to a naive animal (73). Furthermore, in a more relevant model of kidney transplantation in NHP, where tolerance was induced by costimulation blockade combined with donor-specific transfusion (DST), it was revealed that the higher frequency of alloreactive memory cells (when measured by ELISPOST assay) correlated with the occurrence of acute rejection (74).

Collectively, these studies concluded that resistance to the tolerance-inducing effects of costimulation blockade in experimental models and belatacept-resistant rejection in the clinic were caused by heterologous immunity (75, 76). How can this conclusion be reconciled with the recent data showing that effective memory T-cell recall response actually requires CD28 costimulation? One explanation could be that in the non-physiologic context of transplantation, the strength of the antigenic challenge overcomes the costimulation threshold, particularly in Ag-experienced cells.

## An Early and Only Cellular Rejection?

Heterologous immunity occurs through the interaction of a recipient Ag-experienced T cell with a donor APC, in transplant immunology this is called the direct recognition pathway. If we assume that the strength of the antigenic challenge during an allo-immune response overcomes the CD28 requirement threshold, it should again be through the direct recognition pathway. Yet the main immunological issue in kidney transplantation concerns the late onset of kidney dysfunction caused by chronic rejection mainly driven by the indirect pathway (i.e., the interaction of a recipient T cell with a recipient APC exposing donor allogenic MHC peptides) (77), which presumably has a higher physiological CD28 requirement threshold. In addition, the onset of *de novo* DSA can explain a large proportion of chronic rejection. Its onset is dependent on allogenic B-cell response that receives help from a highly specialized subset of CD4 T cells in the germinal center (GC), the follicular helper T cells (Tfh) (78). A recent study revealed that help for a GC alloantibody response could only be provided by CD4 T cells by the indirect pathway (79). The fact that CD28 costimulation is greatly required for primary Tfh response probably explains the lack of DSA in experimental models and belatacept-treated recipients exhibiting remarkably low levels of DSA (64).

The above points suggest that costimulation blockade-resistant rejection should occur early, driven by the direct pathway and consequently without the development of specific alloantibodies, except, obviously, in the case of prior specific immunization.

## Are Experienced-T-Cell Subsets on Equal Terms with Costimulation Blockade Resistance?

Even in cases involving the direct recognition pathway, it is likely that all Ag-experienced T cells are not equal in terms of CD28 requirement. Recent studies on tolerance induction by costimulation blockade (80–85) substantiate the view mentioned above that CD28 requirement loss would not reflect an inherent advantage to any memory response acquired following an immune response, but would be a feature confined to cells reaching the end of a progressive process of maturation.

When allo-specific CD8 T Central Memory (TCM) and T Effector Memory (TEM) cells were transferred into wild-type recipients, they were found equally effective at rejecting allografts. When transferred into aly-deficient recipient (aly-deficiency leads to an absence of secondary lymphoid organs), TEM cells were significantly better than TCM at rejecting allografts (86). This suggests that TCM, but not TEM, reactivation requires the presence of APC with costimulation molecules to proliferate and gain effector and homing capacities.

In line with this, in a model of heterologous immunity generated by a latent γHV68 infection of WT mice, effector T cells (CD44^high^CD127^low^CD62L^low^) and TEM (CD44^high^CD127^high^CD62L^low-int^) were found to be responsible for resistance to tolerance induction by costimulation blockade, in contrast to TCM (80).

In a murine model, decreasing the amount/duration of antigen exposure during priming impacted the ability of donor-specific experienced T cells to mediate costimulation blockade-resistant rejection (81). Interestingly, only donor-specific T cells that were generated under conditions of reduced Ag exposure failed to mediate costimulation blockade (referring to as CD80/86 blockade) resistant rejection. Overall antigenic stimulation undergone by T cells during priming is proposed as predicting cell fate, ranging from unpolarized cells to terminally differentiated cells (87). Thus in the case of poor antigenic stimulation, the accumulation of unpolarized cells could explain the success of the costimulation blockade.

The differential effects of belatacept on cell proliferation in response to either viral peptide processed on self APC or allogenic stimulation seem to confirm this proposition (82). Xu et al. showed that a large percentage of the repertoire proliferated in response to alloantigen, but contained few polyfunctional cells (advanced in their maturation and expressing IFNγ, TNFα, and IL-2). By contrast, the proportion of cells responding to a viral peptide was low and consisted predominantly of mature polyfunctional TEM. When belatacept was added to the cell culture medium, only the more mature cells escaped the costimulation blockade. This again demonstrates that only T cells that have reached a late maturation stage are independent of CD28.

Furthermore, this could explain the relative success of the association of belatacept and alefacept, a CD2 antagonist, in an experimental model of kidney transplantation in NHP (83). Indeed, CD8+CD2+ were the most differentiated in terms of cytotoxic molecule expression and polyspecificity.

Hence among experienced T cells, those liable for costimulation blockade resistance are mature cells, having completed the progressive process of differentiation, including the loss of the CD28 costimulation requirement for their activation.

Interestingly, recent data have revealed that end-stage renal disease patients, compared to healthy controls, have a significant greater number of memory T cells showing progressive terminal differentiation, similar to what is observed in old people with immune-senescence (88). Likewise, anti-thymocyte globulins (ATG)-treated recipient exhibit more late stage differentiated T cells, including CD28 negative (89). Hence, kidney transplant candidates, by definition with impaired renal function, could be especially affected by belatacept-resistant rejection.

Beyond having a CD28 requirement, CTLA-4 might also play a role in belatacept-resistant rejection. Halloran et al. have recently demonstrated that CTLA-4 transcripts dominate the molecular landscape of T-cell-mediated rejection (TCMR) (90), highlighting the possibility that an active negative regulation of T cells in tissue could explain the occurrence of robust TCMR in belatacept-treated recipients.

## CD28 Selective Blockade

Up to now, “CD28 blockade” referred to inhibiting B7, either with a CTLA4-Ig or anti-CD80/86 antibody. Obviously, concomitant inhibition of the CTLA-4 pathway is the main drawback of this strategy. As suggested above, the selective blockade of CD28 signaling (i.e., blocking only CD28/CD80/86 interactions) should present the advantage of respecting the immune-modulatory signals mediated by CTLA-4. The recent development of monovalent antagonist anti-CD28 binders makes this strategy feasible and safe (91–95), clearly differentiating them from agonist or superagonist anti-CD28 antibodies (96–100).

CD28 antagonists prevent acute allograft rejection in mice (101) and primate (92). The potential benefit of preserving CTLA-4 pathways would be due to its extrinsic action, mainly through regulatory T cells. Indeed, use of CD28 antagonist is associated with Treg accumulation in the graft, where they most likely modulate pathogenic T cells and promote prolonged allograft survival (92).

But CTLA-4 has also intrinsic, cell-autonomous roles (102). For experienced T cells, we would expect the advantages of a selective CD28 blockade compared with CTLA4-Ig, if two conditions are met: (i) cells independent of CD28 costimulation for their activation are at play in the context of allo-immune responses and (ii) that activation of these same cells is regulated by CTLA-4. We have seen above that CD28-independent alloreactive cells do exist even though this concerns probably only a few singular cell subsets. Whether experienced T cells are regulated by CTLA-4 is the focus of the following paragraph.

## Are Experienced T Cells Regulated by CTLA-4?

Targeting CTLA-4 with ipilimumab for melanoma immunotherapy was the first clinical demonstration of the physiological role of CTLA-4 acting as an immune checkpoint that controls T-cell reactivity (103, 104). Initial work indicated that the maximal activity of anti-CTLA-4 treatment required the targeting of CTLA-4 on both effectors and Tregs (105). It has also been suggested that anti-CTLA-4 antibodies lead to the depletion of Tregs within the tumor microenvironment in a Fcγ receptor-dependent manner (106–108), concomitant with an increase in the number of activated T cells in peripheral blood (109–112) as well as the tumor site (113–115). Two non-mutually exclusive scenarios can explain this second observation. First, anti-CTLA-4 treatment could improve the priming, then expansion of tumor-specific naive T cells. Second, it could increase the magnitude of the preexisting memory/effector tumor reactive T cells by turning off inhibitory mechanisms (116). Recent advances argue for the latter. Cha et al. measured the frequency of individual rearranged TCRβ genes after anti-CTLA-4 treatment in cancer patients. Clinical outcome was associated with maintenance of high-frequency clones present at the start of the treatment. The bulk of the change in clone frequency occurred in the effector/memory T-cell compartment rather than in the naive T-cell pool, suggesting that treatment boosted meaningful preexisting T-cell responses (117). More recently, it has been evidenced in mice that preexisting anti-tumor T-cell responses are amplified by checkpoint blockade therapy. Anti CTLA-4 and anti PD-1 in a sarcoma model regulated a subset of genes in CD8 tumor-specific infiltrating lymphocyte (TIL) (especially Granzyme B, IFN-γ, and TNF-α that are known to cause acute rejection), whose enhanced expression is similar to that observed in CD8 T cells from mice during acute secondary viral infection. The depressed genes were similar to those of exhausted CD8 T cells in chronic viral infection (particularly LAG-3 and TIM-3) (118). In a melanoma model, anti-CTLA-4 predominantly inhibits Treg cells in TIL but also reinvigorates exhausted PD-1 + Eomes + CD8 T cells (119).

In the context of rejection prophylaxis by CTLA4-Ig, CTLA-4 is also blocked (at least it cannot interact with CD80/86 anymore). It is tempting to speculate that, similar to that which is observed in tumors, some preexisting transplant infiltrating lymphocytes in an advanced stage of differentiation, which are supposed to be costimulation independent, could be reinvigorated by the CTLA-4 blockade with belatacept. Indeed CTLA-4 might inhibit T cells even in the absence of CD28 (120) and data from clinical trials provide indirect evidence for such an “immune checkpoint inhibitory” effect of CTLA4-Ig. In inflammatory bowel disease (IBD), patients treated with CTLA4-Ig demonstrate minimal improvement and disease exacerbation was seen in some treatment groups (121). The development of IBD has also been reported in a patient treated with CTLA-4 Ig for rheumatoid arthritis (122).

## Which Cells are Responsible for Belatacept-Resistant Rejection?

Regardless of maturation stage, we can assume that both the threshold of CD28 requirement and the intrinsic regulation by CTLA-4 pathway differ between various T-cell subsets.

Polarized Th17 could be responsible for Belatacept-resistant rejection, since an elevated level of Th17 memory cells has been associated with acute rejection with belatacept (85), and as mentioned above, in IBD, which is a Th17-mediated disease, CTLA4-Ig treatment has exhibited minimal efficacy and even, in a few cases, disease exacerbation (121). In addition, *Candida albicans* immunization of mice conferred resistance to costimulation blockade following transplantation. *C. albicans* polarizes the response toward Th17 cells and enhances expression of CTLA-4 on Th17 cells. *Mycobacterium tuberculosis*, which polarizes the response toward Th1 cells, does not confer such resistance (84). These data were verified using mice genetically deficient for hallmark T-cell transcription factors such as B6.RORγt KO and B6.T-bet KO (123). Thus, Th17 cells might be particularly sensitive to regulation by CTLA-4, and CTLA4-Ig might hamper this regulation.

Turning to Tfh, the initial priming instigating a Tfh response is CD28 dependent, including in the allo-immune response context (124). By contrast, primed Tfh lose their CD28 requirement when they secondarily interact with B cells (125). Furthermore, at that stage, CTLA-4 also regulates Tfh function in a cell-intrinsic manner (126). Again, like Th17, Tfh accumulates with immune history and has the features required to prompt resistance to CTLA4-Ig.

## Potential Advantage of Targeting CD28 Instead of CD80/86

Recently (127), we performed a direct assessment of FR104 (93), a selective CD28 Fab antagonist, versus CTLA4-Ig (LEA26Y) in kidney allograft in baboon. The biologics were used *de novo* together with an initial 1-month treatment with a low dose of tacrolimus, weaned between months 1 and 2, after which the recipients were under monotherapy with the biologics. Biopsy-proven acute rejection animals were treated with boluses of steroids. In the CTLA4-Ig group (*n* = 5), four out of the five recipients developed severe acute cellular rejection before, during or just after tacrolimus weaning and this proved to be corticoresistant. In the FR104 group (*n* = 5), only two animals developed an acute rejection episode, just after tacrolimus weaning, and this could be reversed by steroids. A transcriptional analysis of 1-month biopsies did not reveal any significant differences except the remarkable exception of IL-21, stronger in CTLA-4 treated animals, whose main source is Tfh cells. Immunohistochemistry revealed some CD4 T cells expressing PD-1, the main marker used to identify Tfh and IL-21. We then assessed *in vitro* proliferation of stimulated Tfh (CXCR5 + ICOS + PD-1 +) using human tonsil tissue and found that inhibition was more effective with FR104 than with CTLA4-Ig. This was confirmed in an experimental model of immunization with KLH in mise where, as expected, primary Tfh response was equally inhibited with both CD28 selective blockade and CTLA4-Ig, unlike the recall response in which the CD28 selective blockade was more efficient in controlling Tfh response. Of interest in a model of islet transplantation, mIL21R.Fc rescues CTLA-Ig-treated mice, resulting in tolerance in 100% of the mice versus 55% in a CTLA4-Ig monotherapy group, and it was demonstrated that IL-21 acted as an antitolerogenic cytokine by preventing Treg generation and inhibiting Treg function (128).

## Summary

In the field of transplantation, the initial great hopes for CD28/CD80/86/CTLA-4 blocking strategy have been dashed in the confrontation with clinical reality. The presence of a complex repertoire of preexisting experienced T cells either free of a CD28 costimulation requirement and/or controlled by the CTLA-4 immune checkpoint is a likely explanation.

However, the picture might not be so dark. First, because primary Tfh response is strictly under the control of CD28, explaining why a costimulation blockade with belatacept prevents the induction of alloantibodies. Second, a loss of a CD28 requirement might not be exhibited by memory cells, but rather confined to terminally matured cells, to some extent exhausted. Although in the context of allo-immune response, these cells could cause severe rejection, the risk of a rescuing inhibitory signal mediated by CTLA-4 and of eliciting belatacept-resistant cellular rejection could be alleviated by the use of CD28-specific antagonists, which are currently in clinical development that will block CD28-mediated signals, without preventing CTLA-4 signals. This novel approach might have the potential advantage of controlling post-transplant immune responses more effectively.

## Conflict of Interest Statement

Nicolas Poirier and Bernard Vanhove are shareholders and employees in Effimune, a company developing CD28 antagonists. Simon Ville and Gilles Blancho have no conflict of interest to declare.
